# *East Wind*, *West Wind*: Toward the modernization of traditional Chinese medicine

**DOI:** 10.3389/fnins.2022.1057817

**Published:** 2022-11-10

**Authors:** Ernesto Yagüe, He Sun, Yunhui Hu

**Affiliations:** ^1^Division of Cancer, Faculty of Medicine, Imperial College London, London, United Kingdom; ^2^The State Key Laboratory of Core Technology in Innovative Chinese Medicine, Tasly Academy, Tasly Holding Group Co., Ltd., Tianjin, China; ^3^Cloudphar Pharmaceuticals Co., Ltd., Shenzhen, China

**Keywords:** network pharmacology, standardization, safety, clinical trials, Parkinson’s disease, Alzheimer’s disease, cancer, cardiovascular disease

## Abstract

Traditional Chinese medicine (TCM) has used herbal remedies for more than 2,000 years. The use of complimentary therapies has increased dramatically during the last years, especially in the West, and the incorporation and modernization of TCM in current medical practice is gaining momentum. We reflect on the main bottlenecks in the modernization of arcane Chinese herbal medicine: lack of standardization, safety concerns and poor quality of clinical trials, as well as the ways these are being overcome. Progress in these areas will facilitate the implementation of an efficacy approach, in which only successful clinical trials lead to the molecular characterization of active compounds and their mechanism of action. Traditional pharmacological methodologies will produce novel leads and drugs, and we describe TCM successes such as the discovery of artemisinin as well as many others still in the pipeline. Neurodegenerative diseases, such as Parkinson’s and Alzheimer’s disease, cancer and cardiovascular disease are the main cause of mortality in the Western world and, with an increasing old population in South East Asia, this trend will also increase in the Far East. TCM has been used for long time for treating these diseases in China and other East Asian countries. However, the holistic nature of TCM requires a paradigm shift. By changing our way of thinking, from “one-target, one-drug” to “network-target, multiple-component-therapeutics,” network pharmacology, together with other system biology methodologies, will pave the way toward TCM modernization.

## Traditional Chinese medicine: Historical perspective and conflict with Western medical practices

In *East Wind: West Wind* American writer and Nobel laureate Pearl S. Buck admirably portrays the conflict between cultures and how our perception and making sense of reality is trapped between tradition and modernity. Through the eyes of her main character, Kwei-lan, married to a Chinese medical doctor educated abroad, the author reflects on the impact Western culture has on Chinese lifestyle. Not alien to this conflict, Pearl. S. Buck, who adopted the Chinese name Sai Zhenzhu, as she spent her early years in China, depicts how Kwei-lan’s cultural upbringing shapes her perception of the world. As biomedical scientists working in the East and the West, we believe a parallel conflict still exists in the incorporation of traditional Chinese medicine (TCM) in Western medical practice. Can the vast knowledge kept in the arcane practices of TCM^1^ be exploited for the discovery and development of novel medicines? We certainly think so, although this will require a great effort in research and acceptance of different ways of thinking by both East and West. Importantly, the development of novel technologies such as systems biology, machine learning and network pharmacology can help us to close the gap between East and West medical practices.

Herbal medicine and acupuncture are the two TCM practices most familiar to Westerners, although TCM encompasses others more exotic such as cupping (heated cup therapy), tuina (massage), qigong (practice of energy circulation), and moxibustion (burnt mugwort therapy) (Cheung, 2011). According to Chinese tradition, all natural phenomena can be categorized into Yin and Yang (the two opposites, complementary and exchangeable aspects of nature). The universe, which consists of five basic elements, wood, fire, earth, metal and water, is constantly moving toward a dynamic balance or harmony (Tang et al., 2008). Although these views can now be seen as awkward and retrograde by Western eyes, certain parallelism is apparent in ancient Greek philosophy. For instance, Empedocles (born 494 BC) considered that the universe was composed of four main elements: fire, air, earth, and water. In TCM, Yin refers to the origin of a process (the “support” that can indeed be material but is not limited to it) and Yang to its manifestation. The constant interplay between Yin and Yang leads to changes in the circulation of energy (Qi) and disturbance of this circulation and that of blood by upsetting the Yin-Yang balance, if unchecked, can be associated to disease. Thus, in Chinese culture the human body is viewed as an entity in equilibrium and TCM aims to restore the harmonious circulation of Qi and the Yin-Yang balance of this complex system using practices developed through thousands of years of empirical testing and refinement (Tang et al., 2008). Once again, certain parallelism can be seen with ancient views of the human body held by Western cultures, such as the four bodily humors in Hippocratic medicine consisting of blood, phlegm, yellow bile and black bile (Kleisiaris et al., 2014). Importantly, Hippocrates also saw disease as the lack of harmony between these four humors. The discerned reader will easily establish parallels between these TCM concepts and well-understood principles of metabolism, immunity, or homeostasis in modern medical science. Both TCM and ancient Western practices are holistic and paid particular attention to the equilibrium and harmony in the body, but modern evidence-based Western medicine has evolved largely based on analytical and reductionist approaches (Evidence-Based Medicine Working Group, 1992). Thus, this conflict between modern Western medicine and TCM arises because of the inherent difficulties both East and West have for appreciating the others’ principles and concepts.

**FIGURE 1:**
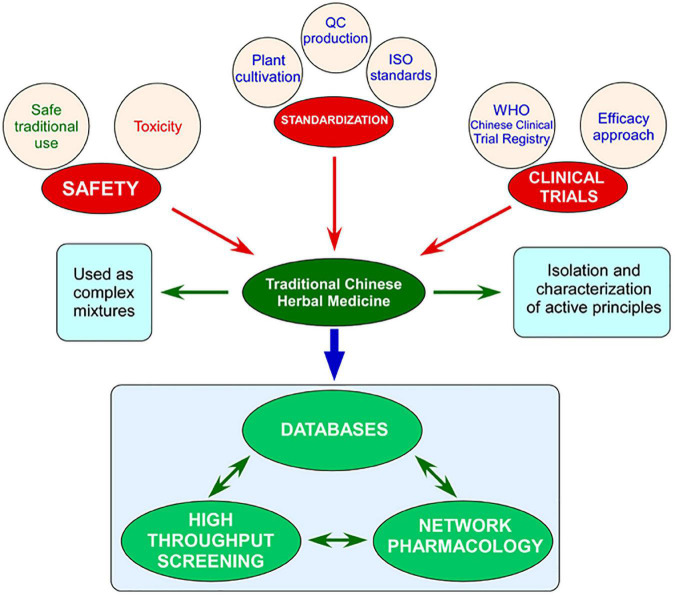
Schematic representation of the main bottlenecks impeding the modernization of Traditional Chinese medicine (TCM) and its incorporation into Western medical practice (safety, standardization, and poor clinical trials) and the ways forward provided by the generation of databases of preparations, compounds, targets; high throughput screening; and network pharmacology.

Several steps are currently being taken to address this conflict. The Chinese government has a long history of supporting the development of TCM. In 2016, The State Council issued the Outline of the Strategic Plan on the Development of Traditional Chinese Medicine (2016–2030), which made TCM development a national strategy and formed part of a wider Healthy China 2030 Plan to improve the health of the Chinese people in the coming 15 years (Wang W. Y. et al., 2021). However, in the era of precision medicine, it is difficult to envision the development of this single strategy, and some commentators have suggested that China should develop its own roadmap in the development of precision medicine taking advantage of the combination of TCM with modern molecular technologies to build a Chinese style precision medicine. This combination of TCM and Western medicine will have to develop methodologies for the classification, diagnosis, treatment and prevention of diseases (Wang and Zhang, 2017). There is no shortage of studies addressing the combination of TCM with modern therapies. To just cite a few, co-administration of Compound Danshen dripping pills and bezafibrate has been found to offer protective effect against diabetic retinopathy (the two drugs may act synergistically) by resisting vascular leakage, increasing retinal thickness and inhibiting inflammation and oxidative stress (Liu et al., 2022); analysis of twenty-one randomized controlled trials with 2,162 patients indicates that the combination of Qishen Yiqi dropping pill with conventional chronic heart failure treatment improves several parameters associated with clinical efficiency (Chen et al., 2021); and *Ginkgo biloba* tablets combined with aspirin improves cognitive impairment in cerebral infarction patients with no dementia (Wang et al., 2015; Table 1). In an effort to close the gap between East and West medical practices, the World Health Organization (WHO) has supported since 2008 the safe and effective integration of TCM within modern Western medicine (Xu et al., 2013) and the European Union Directive 2004/24/EC established the legal and regulatory framework for the use of herbal medicinal products in the EU (Anquez-Traxler, 2011). Hospitals and clinics using a combination of TCM and Western medicine are popular in China and gaining momentum in the West. However, a lot more regulatory and investigative work needs to be undertaken to ensure the consistent safety, efficacy, and quality of TCM. In this review we will offer a personal perspective on the successes and limitation of TCM and how the development of novel omic technologies can pave the way for the integration of TCM in Western medical practice (Figure 1).

**TABLE 1:** Characteristics of main preparations described in the article.

Preparation	Plant components	Use	Active compounds	Effects−Mechanism/s of action	Approval	References
Bushen-Yizhi formula	*Cnidium monnieri, Panax ginseng, Polygonum multiflorum, Cortex moutan, Ligustrum lucidum*, *and Lycium barbarum*	Alzheimer’s disease	138 potential targets identified by network pharmacology	Modulates the cholinergic system and nerve growth factor signaling pathways. Ameliorates oxidative stress and neuronal apoptosis		Cai et al., 2018
Compound Danshen dropping pills	*Radix salviae miltiorrhizae, Radix notoginseng; Borneolum syntheticum*	Coronary heart disease; acute mountain sickness; diabetic retinopathy	Unknown; potential danshensu, savianolic acid B, protocatechuic aldehyde, ginsenosides, notoginsenoside, and borneol	Targets AURKB, MET, and PIM1 kinases; potential synergy with bezafibrate against diabetic retinopathy	Chinese NMPA coronary heart disease and angina pectoris; in clinical use in Australia; United States Phase II chronic angina and acute mountain sickness	Luo et al., 2013; Wang and Zhang, 2017; Liao et al., 2019; Feng et al., 2021; Wang T. et al., 2021
Compound opening arrow mixture (COAM)	>10 herbs	Anti-cancer	Unknown	Activation of caspase 3		Zhou et al., 2020b
Danggui-Shaoyao-san	*Paeonia lactiflora, Angelica sinensis, Ligusticum chuanxiong, Atractylodes macrocephala, Alisma orientale*	Alzheimer’s disease	Ferulic acid, atractylenolide I, ligustilide, tetramethylpyrazine, senkyunolide A, gallic acid, 3-butylphthalide, Z-butylidenephthalide, among many others identified by network pharmacology	Regulation of oxidative stress and inflammation. Target proteins: TSHR, TOP1, CYP3A4, LMNA, and MAPT (Wu et al., 2020); APP, Fas TNFR, VDCC, NMDAR, GluR, SERCA, Tau, NOS, GSK3B, p38, TNFR, Cx I (Luo et al., 2016)		Luo et al., 2016; Wu et al., 2020
Epimedium	*Epimedium brevicornum*	Alzheimer’s disease	Icariin	Improved memory function; decreased amyloid-β peptide and amyloid precursors; enhanced neurogenesis		Li et al., 2015
Fo Shou San (FSS) and Dang gui shao yao san (DGSYS)	*Angelica sinensis*	Neurodegenerative diseases		Ameliorate memory impairment, decrease levels of both precursor and mature amyloid-β in the hippocampus, rescue cholinergic levels, increase activity of superoxide dismutase and decrease malondialdehyde levels. Inhibit expression of inflammatory interleukin 1β and myeloperoxidase in rat models		Wang et al., 2022
Ginkgo	*Ginkgo biloba*	Improve microcirculation, increase cerebral blood flow, improve memory, reduce risk of neurodegenerative disease	Flavonoids and ginkgolides	Anti-oxidant and anti-radical effects		Wang et al., 2015
Huaier	*Trametes robiniophila*	Anti-cancer	Proteoglycan	Down regulation of RAD5, sensitization to radiotherapy; suppression of breast cancer proliferation via linc00339/miR-4656/CSNK2B signaling pathway and by inhibition of cyclin B1 expression, promoting G2/M-phase arrest and modulating the PI3K/AKT signaling pathway	Chinese NMPA alone or combined with other drugs in treatment of various malignancies	Ding et al., 2016; Wang W. et al., 2019; Yao et al., 2020
Jinfukang, Huangqi Guizhi Wuwu decoction and other *Astragalus*-based herbal preparations	*Astragalus membranaceus; Astragalus mongholicus*	Anti-angiogenic and anti-cancer	Formononetin	Suppresses FGF2-triggered activation of FGFR2; inhibits Akt signaling; enhances effect of sunitinib on tumor growth inhibition	Chinese NMPA for non-small cell lung cancer	Cassileth et al., 2009; Wu et al., 2015
Justicia	*Justicia procumbens*	Thrombotic disease	Unknown	Inhibition of platelet aggregation; regulation of F2, MMP9, CXCL12, MET, RAC1, DESA, ABCB1		Hong et al., 2021
LongSengZhi (LSZ) capsule	12 herbs	Cardiovascular disease; heart failure	Unknown	Reduction of ROS; inhibition of inflammation and activation of platelets and endothelial cells		Li et al., 2019; Xu et al., 2020
Lycoris	*Lycoris radiata*	Alzheimer’s disease	Galantamine	Acetyl cholinesterase inhibitor	Licensed in Europe	Li and Zhang, 2009
NaoXinTong (NXT) capsule	16 herbs including *Astragalus membranaceus* and *Salvia miltiorrhiza*	Cardiovascular disease	Unknown; potential amygdalin, paeoniflorin, savianolic acid B, ligustilide, gallic acid, hydroxysafflor yellow A, and butylidenephthalide	Inhibits inflammation, oxidative stress and apoptosis; enhances lipid and glucose metabolism	Chinese NMPA cardiovascular disease	Wang et al., 2017; Han et al., 2019
NeuroDefend (modified Huang-Lian-Jie-Du-Tang)	*Rhizoma coptidis, Scutellaria baicalensis, Cortex phellodendri, Salvia miltiorrhiza, Rhizoma corydalis*, and *Uncaria rhynchophylla*	Alzheimer’s disease		Reduces amyloid-β load	United States provisional patent No.504824619 and United States non-provisional patent No.505236638 for Alzheimer’s disease	Iyaswamy et al., 2020
PHY906	*Scutellaria baicalensis, Glycyrrhiza uralensis, Paeonia lactiflora, Ziziphus jujuba*	Gastrointestinal distress, diarrhea, cramps, nausea, vomiting; anti-tumor	Unknown	Increases capecitabine therapeutic index	Phase II	Kummar et al., 2011; Liu and Cheng, 2012; Changou et al., 2021
Qinghao	*Artemisa*	Malarial relief	Artemisinin, dihydroartemisinin	Alkylation of multiple targets	WHO first line anti-malarial agent	Tu, 2011, 2016; Liu et al., 2014; Wang J. G. et al., 2019
Qishen Yiqi Dripping Pills (QSYQ)	*Astragalus membranaceus, Salvia miltiorrhiza, Panax notoginseng, Dalbergia odorifera*	Heart disease	Unknown; potential formononetin, salvianolic acid B, rosmarinic acid	Cardioprotective effect	Chinese NMPA coronary heart disease and angina pectoris	Ruan et al., 2018; Chen et al., 2021; Zhao et al., 2021
Ruanjian Sanjie decoction	*Pinellia ternata, Prunella vulgaris, Cremastra appendiculata, Sargassum pallidum*	Tumor softening	Unknown	Promotion of apoptosis; activation of caspases 3/7 and 9; downregulation of Bcl2 and survivin		Zhao et al., 2017
Shaoyao Gancao Tang	*Paeonia lactiflora* and *Glycyrrhiza uralensis*	Alzheimer’s disease	Paeoniflorin and ammonium glycyrrhizinate	Reduces amyloid-β aggregation and expression of inflammasome receptors NLRP1 and NLRP3; reduces amyloid-β and Tau in hippocampus and cortex; improves working and spatial memory		Chiu et al., 2021
Tea or drinks with *Dracocephalum moldavica* leaves	*Dracocephalum moldavica*	Alzheimer’s disease	Apigenin, luteolin, acacetin, gardenin B, serophulein, salvigenin, isorhamnetin, tilianin, agastachoside, and kaempferol	Improves memory capacity and inhibits neurodegeneration. Decreases insoluble amyloid β deposition by downregulation of β-secretase; activates nuclear translocation of phosphorylated ERK1/2, leading to an increase in brain-derived neurotrophic factor levels		Liu Q. S. et al., 2018; Chen et al., 2020
Wen-Shen-Yang-Gan decoction (WSYGD)	*Cistanche sp., Paeonia lactiflora, Lindera aggregata, Alpinia oxyphylla, Dioscorea opposita, Uncaria sp.*	Parkinson’s disease	geniposidic acid, coclaurine, rhynchophylline, nootkatone, rutin, echinacoside, acteoside, paeoniflorin, linderane, and quercetin	Ameliorates behavior, slows down reduction of substantia nigra dopaminergic neurons, and reduces neuroinflammatory symptoms in animal models		Zhu et al., 2021
Xiao-Chai-Hu-Tang	*Pinellia ternata, Zingiber officinale, Ziziphus jujuba, Panax ginseng, Scutellaria baicalensis, Glycyrrhiza uralensis*	Anti-cancer	Saikosaponins a, d, and c, wogonin, baicalein, ginsenosides, ephedrine, 6-gingerol, 6-shogaol, glycyrrhizin	Inhibition of PTGS2, NR3C2, CA2, MMP1 and IL-17, TNF, Toll-like receptor, and NF-kB pathways		Zheng et al., 2013; Jin et al., 2021
Xie Gan Wan; Long Dan Xie Gan Wan; Guan Xin Su He	*Aristolochia*	Snake and insect bites; promotion of lactation and urination; reduction of edema	Aristolochic acid	Nephropathies; urothelial cancer; metabolically activated by CYP1A1, CYP1A2, NAD(P)H: quinine oxidoreductase and formation of carcinogens	Banned or heavily regulated in Western countries	Martena et al., 2007; Grollman et al., 2009; Yang et al., 2014
Yishen Huazhuo decoction	*Epimedium sp., Ligustrum lucidum, Psoralea sp., Polygonum multiflorum, Astragalus sp., Ligusticum wallichii, Acorus gramineus*	Alzheimer’s disease	Icariin, tetramethylpyrazine, and triterpenoid saponin	Improves cognitive function; reduction of amyloid-β plaque deposition in the hippocampus		Zhang Y. et al., 2015
Yuanhu	*Corydalis yanhusuo*	Analgesic	Dehydrocorybulbine	Interaction with D2 dopamine receptor		Zhang et al., 2014
Yunnan Dragon’s Blood/Chinese Dragon’s Blood	*Dracaena cochinchinensis*	Parkinson’s and Alzheimer’s disease	4-hydroxy-2,4-dimethoxydihydrochalcone; pterostilbene	Alleviates inflammation		Li et al., 2014

## From preparations to drugs

In TCM each combination of herbs used for treatment is referred to as a preparation. Preparations are cited as early as *circa* 500 BC in *Huangdi’s Internal Classic*, one of the earliest collections of TCM and still regarded as a must-read for all TCM students and practitioners (Cheung, 2011). Many collections have tried to compile all the vast available TCM literature from the last 2,000 years. The largest is *Zhong Guo Ben Cao Quan Shu* (*The Complete Collection of Traditional Texts on Chinese Materia Medica*) comprising 410 volumes and more than 246,000 pages, although some other collections vary considerably in scope and electronic search options (May et al., 2012). By 2008, nearly 400,000 preparations had been described (Stone, 2008). These concoctions may consist of single plants, such as Huaier, the officinal fungus *Trametes robiniophila* which has anti-tumor effects (Ding et al., 2016) or *Epimedium brevicornum* which protects neurons from neurotoxic and ischemic conditions (Li et al., 2015), although more frequently they are complex mixtures of many plants, both cultivated and wild, such as modified Huang-Lian-Jie-Du-Tang, which reduces amyloid-β load in Alzheimer’s disease, and is composed of *Rhizoma coptidis, Scutellaria baicalensis, Cortex phellodendri, Salvia miltiorrhiza, Rhizoma corydalis*, and *Uncaria rhynchophylla* (Iyaswamy et al., 2020; Table 1). These TCMs are frequently, but not always, prepared by hot aqueous extraction (Wang J. G. et al., 2019; Lin et al., 2020; Zhu et al., 2021). The molecular mechanisms by which these preparations act are slowly emerging. For instance, COAM (compound opening arrow mixture) has shown anti-tumor effect in a mouse model of breast cancer by activating caspase 3 (Zhou et al., 2020b); the beneficial effects of LongSengZhi (LSZ) capsule in cardiovascular disease and heart failure can be attributed to the reduction in the production of reactive oxygen species and inhibition of both inflammation and activation of platelets and endothelial cells (Li et al., 2019; Xu et al., 2020), and the flavonoid extract from *Dracocephalum moldavica* (which includes apigenin, luteolin, acacetin, gardenin B, serophulein, salvigenin, isorhamnetin, tilianin, agastachoside and kaempferol) downregulates β-secretase and thus decreases insoluble amyloid β deposition, improving memory capacity and inhibiting neurodegeneration (Liu Q. S. et al., 2018; Table 1). Others, such as Qishen Yiqi Dripping Pills (QSYQ), Fo Shou San (FSS), and Dang gui shao yao san (DGSYS) are much better characterized. QSYQ is composed of *Astragalus membranaceus, Salvia miltiorrhiza, Panax notoginseng*, and *Dalbergia odorifera* and the Chinese National Medical Products Administration (NMPA), formerly the China Food and Drug Administration, approved its use for the treatment of coronary heart disease and angina pectoris in 2003. Analytical techniques identify 87 ingredients within this preparation, although just a combination of 24 of those, such as formononetin, salvianolic acid B or rosmarinic acid, is considered to be bioequivalent to QSYQ, and 15 of them are detected in the plasma of patients after treatment. Molecular targets, either direct or indirect, from some of these components have been well characterized and have been recently reviewed by one of the authors (YH) (Zhao et al., 2021). *Angelica sinensis* is an important component of both FSS and DGSYS, used for neurodegenerative diseases, as these preparations ameliorate memory impairment, decrease levels of both precursor and mature amyloid-β in the hippocampus, rescue cholinergic levels, increase activity of superoxide dismutase and decrease malondialdehyde levels. In addition, they inhibit the expression of inflammatory genes associated with Alzheimer’s disease such as *IL1b* (interleukin 1β) and *Mpo* (myeloperoxidase) in rat models of the disease (Wang et al., 2022). However, in many cases, the mode of action and active compound/s in TCM are not known (Table 1).

One of the best successes of TCM, at least from the Westerner’s viewpoint, is the discovery of artemisinin and its approval by the WHO as a first-line anti-malarial agent (Wang J. G. et al., 2019; Table 1). The use of *Artemisia* (Qinghao) plants in TCM for malarial treatment dates back to *circa* 400 AD, when Ge Hong describes its use for malarial relief in the *Handbook of Prescriptions for Emergency* (*Zhouhou Beiji Fang*) and has formed part of the TCM armamentarium since then. In the West, and after the Second World War, notable progress had been made against the disease, thanks to the development of insecticides, such as DDT, and anti-malarial drugs such as chloroquine. However, despite the WHO’s efforts to globally eradicate malaria, the 1950s and 1960s saw an emergence of DDT-resistant mosquitoes and drug-resistant *Plasmodium* strains. In the late 1960s, and following a Chinese national project on malarial research, Youyou Tu and her team started to screen TCM preparations with the aim of finding novel anti-malarial drugs. After testing more than 2,000 herbal remedies and many drawbacks (i.e., artemisinin becoming inactive if the plant extraction was not performed at cold temperatures), the early 1970s saw successful isolation, determination of chemical structure and clinical trials of artemisinin as well as the development of more potent derivatives, such as dihydroartemisinin (DHA). Successful use of artemisinin in the treatment of malaria in many randomized clinical trials and meta-analyses led to the WHO supporting its use as first-line anti-malarial agent in 2006, to near malaria eradication in the Comoros Islands (Liu et al., 2014), and to the award of the 2015 Nobel Prize in Physiology or Medicine “for her discoveries concerning a novel therapy against malaria” to Tu (Tu, 2016). Although our understanding of the mechanism of action of artemisinin is far from complete, it is well established that artemisinin, a sesquiterpene lactone with a 1,2,4-trioxane moiety as pharmacophore, is a prodrug, rapidly converted to DHA *in vivo*, the activity of which depends on the cleavage of the endoperoxide bridge. Instead of having a single target, the activated drug has over 100 targets, which are alkylated and damaged leading to the disruption of multiple key biological processes and associated toxicity in *Plasmodium* cells. This promiscuity in the association of artemisinin with proteins may be responsible for its success as a drug and for the low frequency of resistant *Plasmodium* strains (Tu, 2011).

Several drugs derived, directly or indirectly, from TCM have been approved by the United States Food and Drug Administration (FDA) (Zhao et al., 2020; Fang et al., 2021). Others, such as galantamine, extracted from the TCM herb *Lycoris radiata*, is a licensed acetyl cholinesterase (AChE) inhibitor for Alzheimer’s disease treatment in Europe (Li and Zhang, 2009; Table 1). As efforts to determine the active compounds in TCM gain momentum, many others will follow in coming years. A notable candidate is formononetin, an isoflavonoid from *Astragalus membranaceus* and *Astragalus mongholicus*, with anti-angiogenic and anti-cancer activities that suppresses FGF2-triggered activation of FGFR2 and Akt signaling, in both breast cancer cells and a xenograft model of breast cancer, and enhances the effect of the VEGFR2 inhibitor, sunitinib, on tumor growth inhibition (Wu et al., 2015). An *Astragalus*-based herbal preparation, Jinfukang, is approved by the China NMPA for the treatment of non-small cell lung cancer (Cassileth et al., 2009). Two other lead molecules, albiflorin and oxymatrine, have been obtained after screening more than 200 TCMs for anti-diabetic compounds. These leads, not only reduce triglycerides content in 3T3-L1 fibroblasts, but show reduced visceral adiposity, glucose intolerance and hepatic steatosis in mice (Zeng et al., 2012). Another well-established preparation is NaoXinTong (NXT) Capsule, which has already been approved by the Chinese NMPA for cardiovascular disease (Table 1). NXT inhibits inflammation, oxidative stress, and apoptosis, while enhancing lipid and glucose metabolism. Importantly, when used at the recovery stage, NXT promotes neovascularization (Wang et al., 2017). Chemical analysis indicates that the preparation contains around 200 chemicals, including amygdalin, paeoniflorin, savianolic acid B, ligustilide, gallic acid, hydroxysafflor yellow A, and butylidenephthalide but it is currently unknown which of these compounds may be responsible for the reported biological activity (Han et al., 2019). Icariin, a flavone derived from *Epimedium brevicornum*, protects neurons from neurotoxic and ischemic conditions as it decreases the amyloid-β peptide and precursor as well as enhancing neurogenesis in a mouse model of Alzheimer’s disease (Li et al., 2015). Yunnan Dragon’s Blood or Chinese Dragon’s Blood from *Dracaena cochinchinensis* alleviates inflammation, which plays a critical role in the pathogenesis on many neurodegenerative conditions, such as Parkinson’s and Alzheimer’s diseases. Different compounds have been characterized from *Dracaena cochinchinensis* extracts using 1D and 2D NMR and mass spectrometry and it has been suggested that 4-hydroxy-2,4-dimethoxydihydrochalcone, the main component in the resin (0.2%), and pterostilbene, are responsible for the therapeutic effect of the preparation (Li et al., 2014; Table 1). Wen-Shen-Yang-Gan decoction (WSYGD), a TCM with positive effects on Parkinson’s disease, ameliorates behavior in rotenone-intoxicated mice, an experimental model recapitulating many features of Parkinson’s disease in humans (Johnson and Bobrovskaya, 2015), slows down reduction of substantia nigra dopaminergic neurons, and reduces neuroinflammatory symptoms (all in mice). Using a combination of purification techniques, such as ultra-performance liquid chromatography and mass spectrometry, 97 peaks were obtained from WSYGD. More importantly, 35 prototype constituents and 27 metabolites of WSYGD after oral administration were detected in mouse plasma. Of these 27, the top 10 constituent are geniposidic acid, coclaurine, rhynchophylline, nootkatone, rutin, echinacoside, acteoside, paeoniflorin, linderane, and quercetin (Zhu et al., 2021; Table 1).

Thus, efforts to isolate and characterize active compounds from TCM preparations, and determination of their mode of action will undoubtedly pave the way for future novel drugs.

## From preparations to therapies

The development of artemisinin as an anti-malarial, and other TCM-derived drugs, has followed a strict western molecular/mechanistic approach. Although we believe this approach should be adhered to, other paths are also worth considering. Jin-Ling Tang from the Chinese University of Hong Kong has suggested that it is imperative to use an “efficacy” approach to study and develop therapies derived from TCM. This would take advantage of the well-established use of a particular TCM preparations (Cheung, 2011) and only proceed further with the molecular characterization of active compounds and their mechanism of action for those showing good efficacy (Tang, 2006). However, the vast majority of clinical trials involving TCM lack rigor (Xu et al., 2013), to the extent that some commentators have considered that, apart from some notable exceptions, TCM is barely effective (Cyranoski, 2018). This state of affairs puts an enormous constraint in the implementation of this approach.

Tang’s efficacy approach is not completely foreign to the West as exemplified with aspirin. The Egyptian Ebers Papyrus, dated back to 1534 BC, already described a willow (*Salix sp.*) bark-derived tonic with anti-inflammatory and pain relief used for non-specific aches and pains. By 216 AD willow was an established remedy in the West. It was not until early 1800s that salicylic acid was isolated from willow bark, leading to the chemical synthesis of acetylsalicylic acid by 1852. The first rigorous clinical trial involving salicylates, published in 1876 in *The Lancet*, found that salicin produced remission of fever and joint inflammation (Maclagan, 1876). Due to its gastric irritation and other unpleasant side effects, Felix Hoffman acetylated the phenol group and obtained acetylsalicylic acid, and numerous trials demonstrated reduction of pain, inflammation, and fever, with no unpleasant side effects. Since 1899 in which the product was registered under the name “Aspirin,” it has been used worldwide as an effective pain reliever and fever reducing agent. However, it was not until the 1970s that John Vane (who shared the 1982 Nobel Prize in Physiology or Medicine in 1982 for “their discoveries concerning prostaglandins and related biologically active substances”) described that this compound resulted in a dose-dependent inhibition of prostaglandin synthesis, and nowadays the mechanism of action of aspirin is well known to be mediated by COX-1 inhibition (Fuster and Sweeny, 2011). Thus, the case of aspirin shows that Western medicine can accept for many years the efficacy of a drug without a mechanistic explanation for its mode of action.

Preparations are complex mixtures (Table 1). PHY906 is a decoction of a mixture of four herbs (*Scutellaria baicalensis*, *Glycyrrhiza uralensis*, *Paeonia lactiflora*, and *Ziziphus jujuba*) used to treat gastrointestinal distress such as diarrhea, cramps, nausea, and vomiting. Phase II clinical trials indicate that PHY906 increases the therapeutic index of capecitabine by enhancing its anti-tumor activity and reduces its toxicity profile in advanced hepatocellular carcinoma (Changou et al., 2021). Although the active compounds in the preparation have not been fully characterized (Kummar et al., 2011), the four herbs act synergistically in a mouse model of colorectal cancer (Liu and Cheng, 2012; Table 1). Anti-dementia TCM preparations have been extensively reviewed, and to cite only two examples, some of the agents in Huang Lian Jie Du Tang (consisting of *Coptis chinensis, Phellodendron amurense, Scutellaria baicalensis*, and *Gardenia florida*), and Tiao Xin Fang (consisting of *Codonopsis pilosula, Poria cocos, Polygala tenuifolia, Acorus tatarinowii*, and *Glycyrrhiza uralensis*), such as genipin, berberine, ursolic acid, syringin, baicalein, glycyrrhizic acid, isoliquiritigenin, and 1-hydroxy-3,6,7-trimethoxy xanthone very likely act cooperatively in combating dementia (Li and Zhang, 2009; Table 1). Although concepts of synergy and antagonism are familiar for the pharmacologist, the idea of complex preparations or mixtures having a more dramatic effect than isolated compounds is a notion that is getting traction in Western science, such as anti-cancer hyperfoods (Veselkov et al., 2019; Gonzalez et al., 2021). Thus, although there will be cases in which a single active molecule may be isolated from a preparation and a molecular mechanism can be attributed to its effects, the chemical complexity of preparations and the synergy between their components complicates the identification of their mechanism of action. Other preparations have a well-established record of traditional use, and, in some, their mechanism of action has started to be unraveled, although no active compounds have been isolated. In cancer, and to cite only a few examples, COAM (a herbal complex mixture with many plants) has anti-tumor effect in a mouse model of breast cancer by activating caspase 3 (Zhou et al., 2020b); Huaier, an officinal fungus, effective in breast cancer treatment (Yao et al., 2020), suppresses breast cancer progression via linc00339/miR-4656/CSNK2B signaling pathway (Wang W. et al., 2019) and sensitizes breast cancer cells to radiotherapy by interfering with the homologous recombination pathway and DNA repair by downregulating RAD51 (Ding et al., 2016); the anti-tumor activity of Ruanjian Sanjie (RJSJ) decoction has been attributed to suppression of the anti-apoptotic proteins BCL2 and survivin, leading to the activation of caspases-3/7 and caspase-9; importantly, administration of RJSJ in combination with doxorubicin is more effective and safer than the chemotherapeutic treatment alone in breast cancer xenografts (Zhao et al., 2017; Table 1). Doxorubicin is widely used as a chemotherapeutic in cancer treatment although it has severe and irreversible cardiotoxicity. Compound Danshen dropping pills (CDDP), a preparation consisting of *Salvia miltiorrhiza*, *Panax notoginseng*, and *Borneolum syntheticum*, ameliorates doxorubicin-induced myocardial fibrosis, inflammation, oxidative stress and apoptosis of mouse cardiomyocytes (Luo et al., 2013). CDDP has been approved by the Chinese NMPA and used since its 1994 market launch in China for treatment of coronary heart disease and angina pectoris, completed Phase II in the United States for chronic angina and approved for clinical use by the Australia Therapeutic Goods Administration, although no active compound has been isolated (Liao et al., 2019; Feng et al., 2021; Table 1). Other preparations used for cardiovascular disease include LSZ Capsule (containing 12 TCM herbs), which reduces reactive oxygen species production and inhibits inflammation, reducing atherosclerosis and thrombosis (Li et al., 2019; Xu et al., 2020) and QiShenYiQi, a TCM preparation containing six herbs with no known active compound, offering cardioprotective effect on transverse aortic constriction-induced heart failure in mice and also approved by the Chinese NMPA for the complimentary treatment of coronary heart disease and angina pectoris in China (Ruan et al., 2018; Table 1). TCM for neurodegenerative diseases has been used for long time in East Asia and has been recently reviewed (Li et al., 2017; Chen et al., 2020). Many TCM preparations for Alzheimer’s disease comprise members of the *Lamiaceae* family. For example, the flavonoid extract from *Dracocephalum moldavica* includes apigenin, luteolin, acacetin, gardenin B, serophulein, salvigenin, isorhamnetin, tilianin, agastachoside and kaempferol. It improves memory capacity by decreasing insoluble amyloid-β deposition due to downregulation of β-secretase and inhibits neurodegeneration by increasing brain-derived neurotrophic factor levels due to nuclear translocation of phospho ERK1/2 (Liu Q. S. et al., 2018; Chen et al., 2020). In Alzheimer’s disease cell and mouse models, Shaoyao Gancao Tang (a TCM composed of *Paeonia lactiflora* and *Glycyrrhiza uralensis*) reduces amyloid-β aggregation and expression of inflammasome receptors NLRP1 and NLRP3, reduces amyloid-β and Tau in hippocampus and cortex, as well as improves working and spatial memory (Chiu et al., 2021; Table 1).

We are thus of the opinion that Tang’s argument on efficacy has merit, especially when preparations show no adverse effects in animal models. Well-designed double-blind, placebo controlled clinical trials, as in the case with PHY906 (Changou et al., 2021), should be encouraged without delay to mitigate the lack of characterization of active compounds or mechanisms of action.

## Safety, standardization, and clinical trials

Although the general public considers that natural products are intrinsically safe, especially when compared to pharmaceuticals, their widely use does not guarantee their safety (Xu et al., 2013). Unfortunately, this erroneous assumption has long been exploited by many complimentary medicine companies and practitioners. The United States National Institutes of Health estimated in 2012 that Americans spent around $30 billion in complimentary health approaches (Nahin et al., 2016). TCM has been developed through millennia of empirical testing and improvement and, in general, side effects of preparations are infrequent and mild. For instance, NTX Capsule, a preparation with cardioprotective effects and approved by the China NMPA for atherosclerosis related cardiovascular disease treatment, produces slight nausea, dizziness, gastrointestinal discomfort, and sour regurgitation in approximately 2% of patients (Han et al., 2019). A meta-analysis of TCM in Parkinson’s disease also indicates that TCM is generally safe, well tolerated and can significantly reduce the side effects of conventional dopamine replacement therapy (Zhang G. et al., 2015). Whilst these and other similar mild side effects in TCM preparations are acceptable for modern Western medicine, others are not, and it has led to the ban of some TCM preparations. An extreme case is exemplified by aristolochic acid, present in preparations composed of *Aristolochia* sp., used in TCM and many other herbal remedies to treat snake and insect bites, promote lactation, urination, and reduce edema (Yang et al., 2014; Table 1). Aristolochic acid has been found to be responsible for Chinese herb and endemic Balkan nephropathies, both associated with high incidence of urothelial (transitional cell) cancer (Grollman et al., 2009). Aristolochic acid is metabolically activated by cytochrome P450 (CYP1A1 and CYP1A2) and NAD(P)H:quinine oxidoreductase, resulting in ultimate carcinogenic species that form adducts with DNA, causing mutations, and neoplastic transformation. Since early 2000s many Western countries have banned or heavily regulated the use of herbal remedies containing aristolochic acid, i.e., Xie Gan Wan, Long Dan Xie Gan Wan, or Guan Xin Su He by the British Medicine and Healthcare Products Regulatory Agency (Martena et al., 2007). However, random sampling by the Dutch Food and Consumer Product Safety Authority in 2007 found 25 preparations still containing important aristolochic acid amounts, such as Mu Tong, Fang Ji, Tian Xian Teng, and Xi Xin. This has fostered research efforts to find detoxification techniques of TCM containing aristolochic acid. Due to its weak acidity, processing with alkaline salts has been suggested as a solution to reduce its levels in preparations. Moreover, advanced modern extraction technologies, such as pressurized liquid extraction and supercritical fluid extraction have also been proposed (Fan et al., 2020). Heavy metals and pesticide contamination of TCM are also areas of particular concern. In a study performed in collaboration between Harvard Medical School, Beijing University of Chinese Medicine, and Hong Kong Baptist University, in which more than 100 herbs used in TCM preparations were analyzed, 69% of samples contained heavy metals and 28% contained pesticides that according to the most conservative consumption scenario could contribute to elevated levels of exposure. Importantly, wild collected plants had higher contaminant levels than cultivated plants (Harris et al., 2011). An excellent example of the methodological approach used for the standardization of PHY906 in clinical trials to maintain interbatch reliability and to establish a chemical fingerprint of the preparation, involving various chromatography techniques as well as mass spectrometry, has been described (Kummar et al., 2011). Safety studies are also critical to rule out unwanted effects of TCM when administered as a co-adjuvant therapy, for instance when used in oncology as a complimentary treatment, as both animal studies and clinical trials indicate that some have synergistic effect with chemotherapeutic regimes (Wu et al., 2015; Zhao et al., 2017; Changou et al., 2021). An important concern about the safety and efficacy of herbal drugs originating from TCM stems from insufficient definitions, problems with identity, purity, and falsifications (Bauer and Franz, 2010). No uniform legal status for these groups of herbal drugs currently exists worldwide, although the European Union has established a legal and regulatory framework for herbal medicinal products (Anquez-Traxler, 2011). This lack of worldwide regulation puts constraints in the monitoring and reporting of adverse TCM reactions, leading to potential risks in drug toxicological research and safety evaluation that are easily ignored. For example, the choice of animal model may have a great influence on the efficacy of drugs, and how to define the approval of animal model is crucial. Although is important to focus on the overall efficacy and clinical safety of TCM, there is a lack of precise analysis and monitoring, including few studies on the pharmacodynamic and toxicological mechanisms. Therefore, there is an urgent need to develop new systematic and holistic quality control methodologies to assess TCM preparations and implementing them in the manufacturing process (Xu et al., 2013; Zhang W. et al., 2019).

Strict pharmacological methodologies have helped in the development of TCM-derived medicines, such as artemisinin, as quality controls are easily implemented when the active compound has been isolated, or the preparation is simple. However, most TCM preparations are complex mixtures, and their active principles, when known, form part of the plant secondary metabolism (Erb and Kliebenstein, 2020). Secondary metabolites comprise many specialized compounds, such as alkaloids, polyphenols including flavonoids, and terpenoids, generally specific for a plant or taxonomic group. They are not strictly necessary for plant growth but required for the plant to survive in its environment (Wink, 2008). Importantly, stress, both environmental and biological, as well as the plant defense response alters notably its secondary metabolism (Isah, 2019). This has important implications for TCM, especially for the use of preparations. Influence of geographical and cultivation conditions, as well as presence of pathogens, will alter the amount of active compounds present in preparations, not to mention the presence of toxins such as pesticides (Stone, 2008). These factors become even more difficult to control when the preparations contain non-cultivated organisms, such as *Sargassum* in RJSJ, although efforts to standardize seaweed growing conditions using aquaculture methodologies are well under way (Garcia-Poza et al., 2020). Preparations vary in potency depending on where and when their constituents were harvested. Moreover, the quality of preparations can vary between manufacturers and from batch to batch (Stone, 2008). Thus, the standardization of methodologies for the cultivation of plants used in TCM, as well as the development of modern quality control techniques (Liu and Cheng, 2012), need to be implemented in order to ensure safe and reproducible materials used in clinical trials and a smooth transition toward the modernization of TCM (Xu et al., 2013). In 2009 the International Organization for Standardization (ISO) created the TCM technical committee ISO/TC 249 focusing on quality and safety of raw materials, manufactured products and medical devices, and of informatics derived from ancient Chinese medicine. In early 2022 up to 77 ISO standards have already been published such as herbal decoction apparatus, determination of heavy metals in herbal medicines used in TCM or the determination of aristolochic acids in natural products by high-performance liquid chromatography, to cite just a few examples (Huang et al., 2020). Sharing one set of standards will help the acceptance of TCM globally, however, this implementation will come at a cost. In addition to its traditional use, one main attractive of TCM in China and SE Asia is its relative low cost, especially when compared to modern, pharmacological therapies. Although part of this economic investment has been taken on by the Chinese government, TCM research investment in 2010 was 4.9 billion Yuan, *circa* US$ 770 million (Cheung, 2011), the increased prices for safe and reproducible TCM preparations and TCM-derived drugs will ultimately be borne by the consumer, both in the East and the West.

An important impediment for the acceptance and integration of TCM in Western medical practice has been the poor quality of clinical trials (Manheimer et al., 2009; Xu et al., 2013; Cyranoski, 2018), although we have previously discussed notable exceptions such as artemisinin (Tu, 2011), FDA-approved TCM-derived drugs (Zhao et al., 2020; Fang et al., 2021) and galantamine (Li and Zhang, 2009). An effort to review the evidence base according to The Cochrane Collaboration, an international not-for-profit organization that prepares and maintains systematic reviews of randomized trials of health care therapies with very rigorous methodological standards in its acceptance of articles, indicated that there was not enough good quality trial evidence in 22 out of 42 herbal TCM reviews to conclude on the efficacy of the evaluated treatment. However, in the remaining 20 reviews there was a suggestion of benefit, qualified by a caveat on poor methodology and heterogeneity (Manheimer et al., 2009). More recently, an overview of Cochrane Systematic Reviews on the harms and benefits associated with all forms of TCM, although most of them (67%) were on Chinese herbal medicine, has been published. This study analyzed 104 Cochrane Systematic Reviews containing 1642 primary studies with 157,943 participants using several descriptive characteristics such as evidence, certainty of findings and methodological quality. The authors found that no definitive conclusions could be drawn from 51.9% of studies, although 40.4% showed some benefit, but with insufficient evidence. Importantly, and regarding only Chinese herbal medicine, diseases such as coronary heart disease, schizophrenia and vascular dementia were those with the best quality of evidence. The authors conclude that the quality of the primary trials must improve (Dai et al., 2022).

Liver cancer is one of the most common cancers and major cause of cancer deaths in China (Zheng et al., 2018) and TCM has been used for more than 2,000 years to treat this disease (Liao et al., 2020). In an effort to quantify the efficacy of Western treatments vs. a combination of Western and TCM treatments for primary liver cancer, 207 studies have been recently analyzed. Importantly, 84% of these studies could not be subjected to statistical analysis due to poor research quality. However, in the remaining 33, a statistical difference was observed indicating enhanced curative effects and improvements in the quality of life. Although promising, the authors acknowledged that larger, stricter trials would be needed in order to draw definitive conclusions (Liu et al., 2021). A similar tone in the discussion on the use of TCM for cardiovascular disease can be seen from the literature. Cardiovascular disease is one of the leading causes of death and disability worldwide and an aging population, both in the West and the East, will increase this trend. A 2016 review of 68 randomized control clinical trials including more than 16,000 patients indicated that TCM associated with significant improvements in surrogate end points for hypertension, coronary heart disease, cardiac arrhythmias, and heart failure, with few, or Western medicine-comparable, side effects. However, the methodological quality of studies was generally poor, and the authors emphasized that larger and better quality trials were needed to ascertain effectiveness with a higher degree of certainty (Hao et al., 2015). A more positive tone is generally seen in trials for neurodegenerative diseases. A 24-week randomized, double-blind, double-dummy, and multicentre clinical trial comparing Yishen Huazhuo decoction (YHD, a complex preparation from 7 herbs containing among many others the flavone icariin, the alkaloid tetramethylpyrazine and triterpenoid saponins, such as several astragalosides, which have been shown to decrease the level of amyloid-β in hippocampus and to improve the spatial learning and memory abilities in animal models) vs. the AChE inhibitor donepezil in Alzheimer’s disease patients indicates that YHD improves cognitive function vs. 5 mg/day donepezil. However, the authors point out several limitations such as small sample size, lack of a group with higher donepezil dose and studying only the Han Chinese population, among others (Zhang Y. et al., 2015; Table 1). For the loss of cognitive functions associated with Alzheimer’s disease a study involving 344 patients tested the effect of conventional therapy (donepezil and/or memantine) with herbal therapy (GRAPE formula, composed of 12 herbs) on cognitive functions tested every 3 months up to 24 months. Compared to conventional therapy alone, conventional therapy in combination with herbal therapy significantly improved scores for cognitive function (Shi et al., 2017). Other current clinical trials for Alzheimer’s disease, including *Ginkgo biloba*, due to its anti-oxidant properties to improve mitochondrial function, in Phase I at Nanjing Medical University have been recently reviewed (Cummings et al., 2021). Several meta-analyses have been performed in the last years to analyze clinical trials for Parkinson’s disease in which traditional therapies were compared to combinations of TCM with traditional therapies. Standard therapies included Madopar, a combination of levodopa and benserazide, a peripheral decarboxylase inhibitor that increases the amount of levodopa crossing into the brain and its subsequent conversion to dopamine (19 trials and 1371 patients) (Wang et al., 2012); dopamine replacement therapy (27 studies, 2314 patients) (Zhang G. et al., 2015); and Madopar or Sinemet, a combination of carbidopa and levodopa (14 high quality clinical trials, 1311 patients) (Shan et al., 2018). Results are promising as UPDRS (Unified Parkinson’s Disease Rating Scale) scores are generally better in the groups treated with a combination of TCM and standard therapy. For instance, three TCMs, Pabing Recipie I, Pabing Recipe III, and Zeng-xiao An-shen Zhi-chan 2 significantly improved Parkinson’s disease symptoms in at least three aspects of the UPDRS scores (Zhang G. et al., 2015). Importantly, TCM alone was not significantly better than placebo (Shan et al., 2018) and all authors are cautious and highlight many limitations in these studies, such as flaws in randomization, placebo and blinding, and concealment, not to mention that Parkinson’s is a complex disease with different types and stages, which can influence response to treatment, and rarely reported. Despite these shortcomings, advances are being made in performing the required large randomized, placebo controlled, blind clinical trials. As part of this progress, the WHO created in 2007 the Chinese Clinical Trial Registry (ChiCTR) as part of its International Clinical Trial Registry Platform (WHO ICTRP) and gave it Primary Registry status. It registers both Chinese and other countries clinical trials in accordance with the WHO ICTRP standards and quality control information and submits data to the WHO ICTRP Central Repository to facilitate the searching of the registry worldwide (Wu et al., 2011; Zhang X. et al., 2019).

## From databases to network pharmacology

The complexity of TCM demands a shift in our ways of thinking such that novel approaches, tools, and methodologies can be developed. In 2008, the “Herbalome Project,” championed by Liang Xinmiao of Dalian Institute of Chemical Physics (Stone, 2008), was launched in China to clarify the chemical composition, structure and function of commonly used Chinese herbs and TCM preparations, to establish a standard resource library, and to interpret the synergistic and complementary mechanism of multiple component in TCM drugs on multiple targets (Xu et al., 2013). Although not without critics (Stone, 2008), its initial phase focused on the development of systematic separation methodologies for resolving and analyzing the complex components in TCM and the establishment of a comprehensive resource library (Zhang et al., 2012). The project uses second-dimensional liquid chromatography, mass spectroscopy and nuclear magnetic resonance spectroscopy for the determination of chemical compositions and multi-property evaluation, including absorption, distribution, metabolism, excretion, and toxicity, together with clinical and systems biology studies for bioactivity tests. A report in 2012 described its progress during phase I (Zhang et al., 2012). Importantly, a novel analgesic lead compound effective against inflammatory pain and injury-induced neuropathic pain, dehydrocorybulbine, has been found in extracts of *Corydalis yanhusuo*, using information gained both through the Herbalome Project and reverse pharmacology. Further characterization indicates that the effect of dehydrocorybulbine effect is primarily due to its interaction with the D2 dopamine receptor without causing antinociceptive tolerance (Zhang et al., 2014; Table 1). Other notable efforts include *TCM Database@Taiwan*, a web-based database containing information on molecular properties and structures of more than 20,000 pure compounds isolated from 453 TCM ingredients. It represents an excellent resource for implementing computer-aided drug design and finding novel lead compounds (Chen, 2011). *TCMAnalyzer*, another web-based toolkit, allows the identification of compounds from a TCM herb, its molecular mechanism at the systemic level and explores potentially targeted bioactive herbs (Liu Z. et al., 2018). A more recent effort combines the systematic characterization and curation of information from databases with novel omics technologies. *HERB*, a high-throughput experiment-and reference-guided database of TCM, links 12,933 targets and 28,212 diseases to 7,263 herbs and 49,258 ingredients, providing pairwise relationships among them (Fang et al., 2021). *HERB* has been used in the determination of the bioactive components in Justicia, an anti-inflammatory TCM used to treat thrombotic disease through inhibition of platelet aggregation, and characterization of its molecular mechanism which involves regulation of F2, MMP9, CXCL12, MET, RAC1, PDESA, and ABCB1 (Hong et al., 2021; Table 1). Other disease-focused databases have already been compiled, such as the *TCMIO* for immuno-oncology (Liu et al., 2020). Further inclusion of proteomic, metabolomic, and meta-genomic datasets will help in the characterization of gene regulatory networks and identification of new types of disease-relevant genes.

The introduction of high throughput screening (HTS) has facilitated drug discovery during the last twenty years. HTS uses automated equipment to examine biological activities in a very large number of samples (up to millions) and biochemical HTS has successfully been used for the discovery of novel anti-diabetic lead drugs from TCM (Zeng et al., 2012). Other similar technologies, such as high-content imaging technology, use image based HTS automated microscopy and image analysis to capture and analyze multiple biological phenotypes simultaneously (Fu, 2021). Using a robust cell-based high-content screening, eight compounds which inhibit renal fibrosis have been identified from a TCM library (Wang et al., 2018). Renal fibrosis is the common final outcome of almost all progressive chronic kidney diseases, promoting scar formation and leading to end-stage kidney failure (Liu, 2011). Importantly, some of these compounds, such as gypenosides, have rarely been described in the literature and may be promising leads for developing novel clinically viable renal anti-fibrotic drugs. The United States National Cancer Institute has generated a library of 664 extracts derived from 332 samples representing 132 distinct TCM plant species which are available in 96 and 384 plates, ready for HTS. In a proof-of-concept study, these extracts have been tested for cytotoxicity in the NCI-60 panel of human tumor cell lines (Shoemaker, 2006) and 3% of the total extracts, representing 8 out of the 132 total species show significant *in vitro* cytotoxicity. Of these, four TCM plant species exhibited significant toxicity in a further 5-dose NCI-60 screening and their active components were identified (He et al., 2019). This is an excellent resource for the screening of compounds present in TCM that should enhance international efforts to systematically evaluate commonly used herb extracts used in TCM. These examples illustrate how guided rational modern drug discovery efforts can help our efforts toward modernization of TCM. But is modernization of TCM just westernization or can the holistic nature of TCM be maintained?

The overall efficacy of TCM is not equal to the efficacy of each individual component in a prescription or the sum of them. In view of the complexity of the chemical components of most TCM preparations, network pharmacology offers novel perspectives to analyze processes such as absorption, distribution through the circulatory system and reaching the target (Hao da and Xiao, 2014). As different components may play their roles through different targets in different diseases, it is necessary to preserve the holistic nature of the components that exert therapeutic effects. Network pharmacology is based on systems biology, polypharmacology, and molecular networks (Goh et al., 2007; Li et al., 2021) and is well suited to analyze relationships between drugs and diseases (Hopkins, 2008). This novel approach is more effective in establishing compound-gene-disease networks than traditional drug discovery pharmacological methodologies and is extremely powerful in the analysis of drug combinations. Importantly, this has led to a paradigm shift from a “one-target, one-drug” to “network-target, multiple-component-therapeutics,” making it a powerful tool suitable for TCM modernization (Zhang R. et al., 2019; Li et al., 2020). Combining *in silico* approaches with experimental data is thus gaining momentum. In colorectal cancer, network pharmacology and molecular docking have recently confirmed the correlation between five core compounds (quercetin, stigmasterol, kaempferol, baicalein, and acacetin) in Xiao-Chai-Hu-Tang, an extract of seven herbs with excellent experimental and clinical results in the treatment of several malignancies, through inhibition of their target molecules (PTGS2, NR3C2, CA2, and MMP1) and signaling pathways (IL-17, TNF, Toll-like receptor, and NF-κB) (Zheng et al., 2013; Jin et al., 2021; Table 1). Also in a recent study, one of the authors (YH) took CDDP as a study case to obtain its direct pharmacological targets. Thirty potential kinases were initially considered, with nine of them showing potential dose-dependent effects and three of them, AURKB, MET, and PIM1, being validated at biochemical and cellular levels (Wang T. et al., 2021). Network pharmacology has been used to study the ten most used TCM herbs, including *Ginkgo biloba* and *Huperzia serrata*, for Alzheimer’s disease. Ten thousand and sixteen compounds were obtained (top clusters included steroids, unsaturated fatty acids/alkyls, bridge hydrocarbons and flavones/trimethoxyxanthrones). Target prediction was used to construct compound-target and target-pathway networks. Twenty-four targets clustered into three biological processes groups (learning or memory; negative regulation of synapsis; serotonin receptor signaling) with molecular functions in three categories: β-amyloid binding, serotonin receptor activity and extracellular ligand-gated ion channel activity. This will be useful to gain insights into mechanism of action of anti-Alzheimer’s disease TCM, although the study did not perform any experimental validation (Fang et al., 2017). Bushen-Yizhi formula (BSYZ, made with *Cnidium monnieri* fruits, *Panax ginseng*, *Polygonum multiflorum*, *Cortex moutan*, *Ligustrum lucidum*, and *Lycium barbarum* fruits) exerts its anti-Alzheimer’s disease effects by modulating the cholinergic system and nerve growth factor signaling pathways. It ameliorates oxidative stress and neuronal apoptosis *in vivo*, protecting from scopolamine-induced cognitive impairment. It also improves cognitive dysfunction through the SIRT1/endoplasmic reticulum stress pathway in aging mice. *In silico* analysis highlighted 329 candidate compounds, which were further reduced to 138 Alzheimer’s disease-related targets. Multiple network analysis, including compound-target and target-function analyses, followed by experimental validation demonstrated the therapeutic effects of BSYZ on cognitive dysfunction in APP/PS1 mice (a double transgenic mice with neurons expressing a chimeric mouse/human amyloid precursor protein and a mutant human presenilin 1; both mutations are associated with early-onset Alzheimer’s disease), possibly via regulating amyloid-β metabolism and suppressing neuronal apoptosis (Cai et al., 2018; Table 1). Using systems pharmacology, 19 *Evodia rutaecarpa* potential active components for Alzheimer’s disease have been identified, being the alkaloids evodiamine and berberine those with the highest oral bioavailability. Using a compound-target pathway network analysis of both alkaloids, muscarinic receptors have been suggested to be targets in Alzheimer’s disease as they regulate various sensory, cognitive, and motor functions. However, the study did not offer any experimental validation (Fang et al., 2020). The importance of experimentally validating network pharmacology results is of paramount importance, as illustrated by two studies on Danggui-Shaoyao-san (DSS), a TCM which alleviates Alzheimer’s disease symptoms in animal experiments and clinical studies (Wu et al., 2020), as two different studies highlight completely different set of target proteins (Luo et al., 2016; Wu et al., 2020). This highlights some of the current limitations of network pharmacology, as the use of different algorithms may lead to different predicted results (Luo et al., 2020). In addition, data on various drugs, genes, proteins, and preparations are not comprehensive and computer network screening used for target prediction may not have enough experimental support (Zhou et al., 2020a). It has also been noted that network pharmacology is limited by the fact that it mines existing databases, which are not comprehensive, for known pathways and biological processes, restricting the discovery of novel targets (Chandran et al., 2017; Yuan et al., 2022). Databases show also discrepancies due to numerous sources of information, theoretical and experimental data used in their generation (Chandran et al., 2017). Lastly, ADMET (absorption, distribution, metabolism, excretion, and toxic effects) profiling is necessary to validate active compounds’ pharmacokinetic properties (Noor et al., 2022). Although the use of network pharmacology in TCM studies is still in its infancy (Zhou et al., 2020a), we are of the opinion that this and other system-based technologies together with omics and TCM databases will help in shifting the paradigm necessary for the modernization of TCM, while maintaining its holistic viewpoint and facilitate its incorporation in Western medical practice.

## Concluding remarks

Modernization of TCM requires improvements in standardization, safety and, very importantly, clinical trials. These will facilitate the implementation of an efficacy approach and pave the way toward the acceptance in the West of many preparations. Traditional pharmacological methodologies will reveal novel leads, especially from simple preparations. However, complex preparations will benefit from the development of comprehensive databases, systems biology methodologies, and network pharmacology. This paradigm shift will contribute to the exploitation of the vast knowledge hidden for more than 2,000 years in Chinese herbal medical practice.

## Author contributions

EY investigated the data, wrote the original draft of the manuscript, and wrote, reviewed, and edited the manuscript. HS carried out the funding acquisition and project administration, and wrote, review, and edited the manuscript. YH performed the conceptualization, investigated the data, and wrote, reviewed, and edited the manuscript. All authors contributed to the article and approved the submitted version.

## Footnotes

1 For most of this article, the authors use TCM as a synonym of traditional Chinese herbal medicine.
